# DNA damage stress-induced translocation of mutant FUS proteins into cytosolic granules and screening for translocation inhibitors

**DOI:** 10.3389/fnmol.2022.953365

**Published:** 2022-12-20

**Authors:** Masahiro Nogami, Osamu Sano, Keiko Adachi-Tominari, Yoshika Hayakawa-Yano, Takako Furukawa, Hidehisa Iwata, Kazuhiro Ogi, Hideyuki Okano, Masato Yano

**Affiliations:** ^1^Innovative Biology Laboratories, Neuroscience Drug Discovery Unit, Research, Takeda Pharmaceutical Company Limited, Fujisawa, Japan; ^2^Shonan Incubation Laboratories, Research, Takeda Pharmaceutical Company Limited, Fujisawa, Japan; ^3^Department of Physiology, School of Medicine, Keio University, Tokyo, Japan; ^4^Division of Neurobiology and Anatomy, Graduate School of Medical and Dental Sciences, Niigata University, Niigata, Japan

**Keywords:** FUS, stress granule, amyotrophic lateral scelerosis, compound library screening, RNA-binding protein

## Abstract

Fused in sarcoma/translated in liposarcoma (FUS) is an RNA-binding protein, and its mutations are associated with neurodegenerative diseases, including amyotrophic lateral sclerosis (ALS), through the DNA damage stress response, aberrant stress granule (SG) formation, etc. We previously reported that translocation of endogenous FUS into SGs was achieved by cotreatment with a DNA double-strand break inducer and an inhibitor of DNA-PK activity. In the present study, we investigated cytoplasmic SG formation using various fluorescent protein-tagged mutant FUS proteins in a human astrocytoma cell (U251) model. While the synergistic enhancement of the migration of fluorescent protein-tagged wild-type FUS to cytoplasmic SGs upon DNA damage induction was observed when DNA-PK activity was suppressed, the fluorescent protein-tagged FUS^P525L^ mutant showed cytoplasmic localization. It migrated to cytoplasmic SGs upon DNA damage induction alone, and DNA-PK inhibition also showed a synergistic effect. Furthermore, analysis of 12 sites of DNA-PK–regulated phosphorylation in the N-terminal LC region of FUS revealed that hyperphosphorylation of FUS mitigated the mislocalization of FUS into cytoplasmic SGs. By using this cell model, we performed screening of a compound library to identify compounds that inhibit the migration of FUS to cytoplasmic SGs but do not affect the localization of the SG marker molecule G3BP1 to cytoplasmic SGs. Finally, we successfully identified 23 compounds that inhibit FUS-containing SG formation without changing normal SG formation.

**Highlights**

1. Characterization of DNA-PK-dependent FUS stress granule localization.
2. A compound library was screened to identify compounds that inhibit the formation of FUS-containing stress granules.

## Introduction

Control of the integration and localization of macromolecular machines is essential for normal cellular function (Spannl et al., 2019). These functional assemblies of large molecules are now broadly categorized as organelles with lipid bilayer membranes and membraneless organelles (Gomes and Shorter, 2019). In particular, most membraneless organelles, which are called RNP granules, consist of nucleic acids, RNA, and RNA-binding proteins (RBPs). There are many types of RNP granules, and many RBPs have the intrinsic ability to promote the formation of large molecular assemblies through the process of liquid–liquid phase separation (LLPS; Mateju et al., 2017). Among these assemblies, stress granules (SGs), which are cytoplasmic granules formed under stress conditions, are a normal cellular defense against intracellular and environmental stresses and contain many cytoplasmic types of RBPs, translation control factors, etc., thereby repressing general mRNA translation during cellular stress (Maziuk et al., 2017; Markmiller et al., 2018; Wolozin and Ivanov, 2019). On the other hand, nuclear-localized RBPs do not necessarily localize to SGs during the cellular stress response but have been reported to migrate to SGs in response to various stimuli. Extensive studies have indicated that SGs are transient RNPs, but chronic stresses associated with aging lead to irreversible SG formation related to pathological protein aggregation (Gao et al., 2017; Wolozin and Ivanov, 2019). Posttranslational modification of RBPs is strongly involved in the RBP translocation and SG formation (Wolozin and Ivanov, 2019). Interestingly, a nucleus-enriched RBP, TDP-43, translocates to cytoplasmic SGs in response to some stimuli and has been identified as a core component of pathogenic protein aggregation, called “TDP-43 proteinopathy” in amyotrophic lateral sclerosis (ALS), Huntington’s disease, Alzheimer’s disease, and FTD (Arai et al., 2006; Hasegawa et al., 2008; Tada et al., 2012; Ling et al., 2013). In addition, FUS, TIA-1, hnRNPA2B1, and hnRNPA1, which are also nuclear RBPs, are closely related to pathological protein aggregation in ALS and FTD (Neumann et al., 2009; Kim et al., 2013; Ling et al., 2013; Mackenzie et al., 2017; Wolozin and Ivanov, 2019). These pathological aggregates of nuclear-enriched proteins are often observed in the cytoplasm. Therefore, the molecular mechanism underlying the cytoplasmic-nuclear shuttling activity of the causative RBPs has attracted attention as a cause of ALS pathology. The C-terminal region of FUS, called the noncanonical R/H/KX2-5PY NLS, has sufficient nuclear import activity. Mutations in this C-terminal region have been identified in many patients with familial FUS-ALS (Lee et al., 2006; Belzil et al., 2009; Chiò et al., 2009; Kwiatkowski et al., 2009; Vance et al., 2009). Furthermore, it has been reported that the NLS activity of FUS mutant proteins, which is involved in the cytoplasmic-nuclear localization of these proteins, correlates with a decrease in the age at disease onset (Dormann et al., 2010).

Other groups and we have been establishing an *in vitro* motor neuron model of ALS and analyzing its molecular mechanisms using iPS cell technologies (Higelin et al., 2016; Ichiyanagi et al., 2016; Fujimori et al., 2018; Matsuo et al., 2021). These disease models are extremely useful tools not only for analysis of the molecular mechanisms of disease but also as a biologically relevant tool for small molecule compound library screening of human diseases (Okano et al., 2020; Okano and Morimoto, 2022). In particular, we established a familial ALS disease model by establishing iPS cells from a family with a mutation in the *FUS* gene and isogenic iPS cells with the same mutation. In differentiated motor neurons, the FUS protein exhibits slight cytoplasmic leakage in FUS^H517D^-mutated cells than in control cells, and arsenite treatment causes mislocalization of FUS^H517D^ into G3BP1-positive SGs. In contrast, wild-type FUS is not mislocalized (Ichiyanagi et al., 2016). Furthermore, in our iBRN analysis, a method that classifies the molecules with a substantial impact on transcriptomic data in FUS^H517D^ mutant cells, we identified *PRKDC*, *TIMELESS*, *miR125b*, etc. using cellular models of familial FUS-ALS (Nogami et al., 2021a). All three molecules contribute to a common pathway in the DNA damage response, and *PKRDC*, encoding DNA-dependent protein kinase (DNA-PK), has been reported to be involved in hyperphosphorylation of the FUS protein and to have a mitigating effect on LLPS of the FUS protein *in vitro* (Deng et al., 2014; Murray et al., 2017). Furthermore, we found that in addition to DNA damage induction, cotreatment with inhibitors of DNA-PK resulted in translocation of endogenous wild-type FUS into SGs (Nogami et al., 2021a). Similar studies have also indicated that mutations in the FUS NLS induce an abnormal poly-ADP-ribose polymerase (PARP)-dependent DNA damage response that is responsible for neurodegeneration and FUS mislocalization and aggregate formation (Naumann et al., 2018). Therefore, DNA damage response signaling due to the aberrant nucleocytoplasmic shuttling of the FUS protein is believed to be an upstream molecular target for ameliorating FUS-ALS pathology.

Here, we performed detailed experiments on FUS-containing cytoplasmic SG formation using various pathogenic FUS mutants and FUS with mutations in the DNA-PK–regulated phosphorylation site further to explore the molecular mechanisms of FUS-positive SG formation. We observed a synergistic effect of the DNA damage inducer CLM and the DNA-PK inhibitor NU7441 on the translocation of FUS into SGs. We confirmed that this synergistic effect was dependent on the activity of DNA-PK. Furthermore, we screened for small compounds that specifically inhibited the formation of only FUS-positive SGs without affecting the formation of FUS-negative SGs and succeeded in identifying essential compounds, including signal transduction-inhibiting and chromatin-related molecules.

## Materials and methods

### Vector construction and plasmid preparation

Vector construction was performed by a service provider (GENEWIZ, South Plainfield, NJ). The synthesized Venus-human FUS^WT^, Venus-human FUS^H517D^, Venus-human FUS^P525L^, and mCherry-human G3BP1 sequences were subcloned into the *Hin*dIII/*Not*I sites in the pcDNA3.1(+) vector (Invitrogen, Carlsbad, CA) to obtain the corresponding expression vectors with N-terminal fusion tags. In addition, the synthesized Venus-human FUS^WT-Ala^ and Venus-human FUS^WT-Asp^ sequences were subcloned into the *Bam*HI/*Xho*I sites in the pcDNA3.1(+) vector to obtain pcDNA3.1(+)-Venus-human FUS^WT-Ala^ and pcDNA3.1(+)-Venus-human FUS^WT-Asp^, respectively. To obtain expression vectors for Venus-FUS^P525L-Ala^ and Venus-FUS^P525L-Asp^, mutagenesis of pcDNA3.1(+)-Venus-human FUS^WT-Ala^ and pcDNA3.1(+)-Venus-human FUS^WT-Asp^ was performed to replace Pro with Lys at amino acid position 525 in the FUS protein.

### Cell culture and transfection

The human astrocytoma cell line U251 MG (KO) was purchased from the JCRB Cell Bank (Tokyo, Japan). U251 MG (KO) cells were cultured in E-MEM containing L-glutamine, phenol red, sodium pyruvate, nonessential amino acids, and 1,500 mg/l sodium bicarbonate (Wako, Osaka, Japan; #055–08975) supplemented with 10% fetal bovine serum (Life Technologies; #10437085) and 100 U/ml penicillin–streptomycin (Life Technologies; #15140122) under 5% CO^2^ at 37°C. To obtain cells stably overexpressing Venus-tagged FUS and mCherry-tagged G3BP1, the appropriate expression vectors were introduced into cells with Lipofectamine 3000 (Life Technologies) in Opti-MEM I (Thermo Fisher Scientific, Waltham, MA; #31985070) following the manufacturer’s instructions. U251 MG (KO) cells positive for Venus and mCherry signals were selected with 100 μg/ml G418 (geneticin; Thermo Fisher Scientific). To induce DNA damage, cells were treated with 10–100 nM CLM (MedChem Express, Monmouth Junction, NJ; #HY19609) with or without 1–10 μM NU7441 (Wako; #143-09001), a high-potency selective DNA-PK inhibitor.

### Western blotting

Western blotting was performed as described in the previous study with slight modification (Nogami et al., 2021b). U251 MG (KO) cells were collected into 1× SDS Blue loading buffer (NEB, Tokyo, Japan; #B7703S) and heated at 95°C for 5 min. Next, genomic DNA in the samples was sheared using a syringe needle (Terumo, Tokyo, Japan; #SS-05 M2913). Electrophoresis was performed with TGX AnyKD gels and 7.5% gels (Bio-Rad; #4569036), and proteins were transferred with a Transblot Turbo blotting system PVDF PAK MINI (Bio-Rad, Hercules, CA; #1794156). The protein-containing membranes were blocked with 5% nonfat dry milk (Cell Signaling Technology; #9999S) in Tris-buffered saline containing Tween 20 (TBST; Cell Signaling Technology; #9997S) for 30 min at room temperature. The membranes were then incubated with mouse monoclonal anti-phospho-histone H2A.X (Ser139; Millipore; #05–636), rabbit polyclonal anti-TDP-43 (Proteintech, Chicago, IL; #1078-2-AP), mouse monoclonal anti-FUS (Santa Cruz Biotechnology, Santa Cruz, CA; #SC-47711), and anti-β-actin (Novus Biologicals, Littleton, CO; #NB600-532) primary antibodies in blocking buffer overnight at 4°C. After three washes with TBST, the membranes were incubated with horseradish peroxidase-conjugated anti-mouse IgG (Cell Signaling Technology; #7076P2) or horseradish peroxidase-conjugated anti-rabbit IgG (Cell Signaling Technology; #7074P2) secondary antibodies in TBST for 30 min at room temperature. Luminescence signals on the membranes were detected by an image reader (LAS-4000 system; Fujifilm, Tokyo, Japan) with SignalFire ECL Reagent (Cell Signaling Technology; #6883S). The acquired images were processed with Multi Gauge version 3.1 software equipped with the LAS-4000 system and iBright CL1000 (Invitrogen).

### Immunofluorescence microscopy

U251 MG (KO) cells were cultured in noncoated Cell Carrier 96-well plates. After washing with phosphate-buffered saline (PBS; Wako; #166–23,555), the cells were fixed with 4% paraformaldehyde/phosphate buffer solution (Wako; #163–20,145) for 15–30 min on ice and permeabilized by three incubations with 0.1% Triton X-100 in high-salt buffer (500 mM NaCl, 1 mM NaH_2_PO_4_∙2H_2_O, 9 mM Na2HPO4, and 0.1% Tween 20) for 10 min at room temperature. The cells were then treated with 1% bovine serum albumin (Sigma; #A7030-100G) for 30 min at room temperature and incubated with mouse monoclonal anti-phospho-Histone H2A.X (Ser139; Millipore; #05–636), mouse monoclonal anti-FUS (Santa Cruz Biotechnology; #sc-47711; diluted 1:250), rabbit polyclonal anti-G3BP1 or mouse monoclonal anti-G3BP (Bethyl Laboratories, Montgomery, TX; #A302-033A; diluted 1:250, BD Transduction #611127 diluted 1:500), rabbit polyclonal anti-TDP-43 (Proteintech; #1078-2-AP; diluted 1:250), anti-Flag-M2 (Sigma F3165 1:500), anti-QKI5 (Bethyl Laboratories; diluted 1:500), anti-GFP (Proteintech, #50430-2-AP 1:250 & Rockland #600–101-215 1:250) primary antibodies in low-salt buffer (0.05% Tween 20 in PBS) overnight at 4°C. After three 5-min washes with high-salt buffer at room temperature, the cells were incubated with Alexa Fluor 488 goat anti-rabbit IgG (H + L; Invitrogen; #A11034) and Alexa Fluor 594 goat anti-mouse IgG (H + L; Invitrogen; #A11032) secondary antibodies in a low-salt buffer for 30 min at room temperature. After three 5-min washes with high-salt buffer at room temperature, immunofluorescence signals were observed under a fluorescence microscope (Keyence, Osaka, Japan; BZ-X710 and BZ-X810) equipped with a 20× (Nikon, Tokyo, Japan; PlanApo l, NA = 0.75) or 40× (Nikon; PlanApo l, NA = 0.95) objective lens, and images were acquired with the equipped software (BZ-X Viewer and Analyzer).

### Measurement of Venus-positive granule signal intensities

Images were acquired with an IN Cell Analyzer 6000 system (GE Healthcare Japan) using a 40× objective lens. The intensities of Venus fluorescence signals in cytosolic SGs or nuclear granules were calculated from the acquired images using IN Cell Developer Toolbox software (GE Healthcare Japan).

The precise method used for quantification was as follows.

Step 1: “Nucleus” segmentation using images of Hoechst staining. The “nuclear center” was predefined as a seed region in the nucleus with the objective segmentation module and postprocessing nodes, such as erosion and sieving. The “nucleus” was defined as the nuclear region corresponding to the “nuclear center” with the objective segmentation module. Postprocessing nodes, such as clump breaking with the “nuclear center,” erosion, sieving, and border object removal, were used to separate spatially close nuclei and properly segment the diverse nuclear phenotypes.

Step 2: “Cell” segmentation using images from the Venus channel. The “cell” was defined as the cellular region with the objective segmentation module, with high sensitivity to detect weak signals. Postprocessing nodes, such as erosion, clump breaking using the “nucleus,” sieving, and filling holes, were used to separate spatially close cells adequately.

Step 3: “Granule” segmentation. The Venus-FUS fluorescence images were used to detect textures, such as granules and vesicles, which were recognized by the vesicle segmentation module. The “cell” and “nucleus” were linked with the “granules” using a one-to-many target linking approach to quantify the density × area of “granules” in each cell.

Step 4: Measurement nodes. After individual cells were segmented, the total vesicle intensity and nuclear vesicle intensity per cell were calculated. The cytosolic vesicle intensity per cell was calculated using the following equation: “Cytosolic vesicle intensity per cell” = “total vesicle intensity per cell” – “nuclear vesicle intensity per cell.”

### Compound screening

The human astrocytoma cell line U251 MG (KO) was grown in DMEM (high glucose, GlutaMAX Supplement, containing pyruvate; Life Technologies, #10569–010) supplemented with 10% FBS (Corning, #35-076-CVR) and penicillin–streptomycin solution (Wako, #168–23,191) and maintained at 37°C in 5% CO_2_. mCherry-G3BP1-and Venus-FUS^P525L^-expressing U251 MG (KO) astrocytoma cells were seeded separately in a Cell Carrier-384 Ultra Plate (6057302) at 3000 cells/well and incubated in a CO_2_ incubator at 37°C overnight. Approximately 8,000 compounds were tested at a single concentration (3 μM) on the 1st screen. For the 2nd screen, 303 compounds adjacent to the 61 hit compounds identified in the 1st screen were tested at four concentrations (10, 3, 0.3, and 0.03 μM). After the cells were treated with the compounds overnight, calicheamicin (CLM; 100 nM; MedChem Express, #HY-19609/CS-5320) and NU7441 (1 μM, Wako, #149–09003) were added simultaneously and incubated in a CO_2_ incubator at 37°C for 3.5–5 h. Then, the cells were fixed with 4% PFA (containing Hoechst 33258 or Hoechst 33342) and washed with PBS. Images were acquired using an IN Cell Analyzer 6,000 (GE Healthcare Japan) with a 40x objective lens. Granular Venus fluorescence signals in the cytoplasm or nucleus were analyzed with the IN Cell Developer Toolbox (GE Healthcare Japan). The intensity of nuclear granules was quantitated as “intensity of all granules” - “intensity of nuclear granules.”

## Results

### Subcellular localization of Venus-tagged FUS^WT^ and mutated FUS proteins in response to DNA damage stress

**Figure 1:**
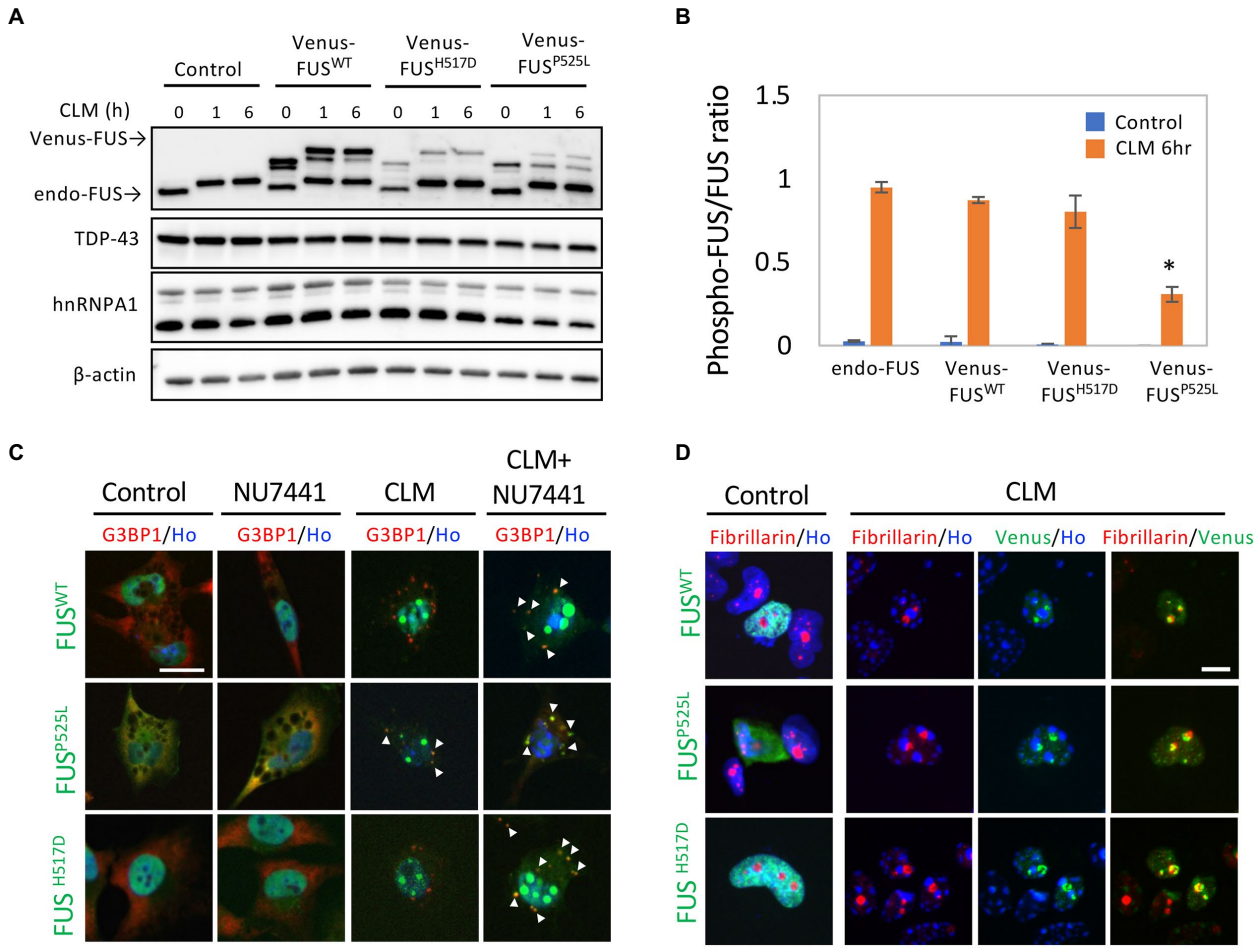
Subcellular localization of Venus-tagged FUS^WT^ (Venus-FUS^WT^) and mutated FUS proteins in living cells. **(A)** U251 MG (KO) cells overexpressing Venus-FUS^WT^, Venus-FUS^H517D,^ and Venus-FUS^P525L^ were generated, and Western blotting with an anti-FUS antibody was performed to confirm the expression of Venus-tagged FUS. Cells were treated with 100 nM calicheamicin (CLM) for 1 and 6 h. An anti-”TDP-43/hnRNPA1/β-actin” antibodies were used as a control. **(B)** Quantification of ratio between phosphorylated FUS associated with band shift and normal molecular weight FUS band in endogenous and Venus-FUS^WT^, Venus-FUS^H517D^ and Venus FUS^P525L^ at the normal and CLM 6 h treatment with the Image J. The migration of Venus-FUS^P525L^ protein shows significantly lower compared to other FUS variants by CLM stimulation. Asterisk indicates significant change from three independent experiments (*t*-test *p* < 0.001). **(C)** The subcellular distribution of Venus-FUS^WT^, Venus-FUS^H517D,^ and Venus-FUS^P525L^ (Green) co-stained with G3BP1 (Red), a marker for SGs and Hoechst (Blue), a marker for nuclei was observed in cells by fluorescence microscopy. Cells were treated with the DNA-PK inhibitor NU7441 (1 μM) for 3 h and were then treated with 100 nM CLM or DMSO (control) for 6 h in the presence of 1 μM NU7441 or DMSO (control). Arrowheads indicate G3BP1 positive cytosolic SGs with Venus-FUS^WT^ or Venus-FUS mutant proteins. Scale bar: 20 μm. **(D)** The Venus-FUS^WT^, Venus-FUS^H517D^ and Venus-FUS^P525L^ protein were colocalized with the nucleolar marker fibrillarin in a CLM-dependent manner by CLM stimulation for 6 h. Scale bar: 10 μm.

To investigate the change in the subcellular localization of mutated FUS proteins in response to DNA damage stress, we generated U251 MG (KO) cells that stably expressed FUS^WT^ (Venus-FUS^WT^) and mutated FUS proteins tagged with Venus, an improved yellow fluorescent protein (Nagai et al., 2002), to observe the effects of DNA damage stress. As expected in our previous study (Nogami et al., 2021a), treatment with calicheamicin (CLM) induced shifts in the Venus-FUS^WT^ and Venus-FUS^H517D^ protein bands in addition to endogenous FUS at high molecular weights, indicating that hyperphosphorylation of these proteins in response to DNA damage stress. In contrast, the Venus-FUS^P525L^ protein was not phosphorylated well, and its nonphosphorylated form remained after CLM treatment (Figures 1A,B). These results indicated that the Venus-FUS^P525L^ protein had a lower ability about 30% to undergo phosphorylation by DNA-PK than the Venus-FUS^WT^ and Venus-FUS^H517D^ proteins (Figure 1B). We next investigated the subcellular localization of the Venus-FUS^WT^, Venus-FUS^H517D^, and Venus-FUS^P525L^ proteins by microscopy. In the absence of DNA damage stress, the Venus-FUS^WT^ and Venus-FUS^H517D^ proteins were mainly localized in the nucleus, while the Venus-FUS^P525L^ protein was distributed not only in the nucleus but also in the cytosol, similar to G3BP1 protein, known as a cytosolic RBP (Figure 1C). Upon CLM treatment, the Venus-FUS^WT^ and Venus-FUS^H517D^ proteins remained in the nucleus, while the Venus-FUS^P525L^ protein was translocated into cytosolic SGs, co-labeled with G3BP1 protein as a marker for cytosolic SGs (Figure 1C). After cotreatment with CLM and NU7441, an inhibitor of DNA-PK, which is known to be a kinase of FUS (Deng et al., 2014), the Venus-FUS^WT^, Venus-FUS^H517D^ and Venus-FUS^P525L^ proteins were obviously translocated into cytosolic SGs (Figure 1C). Importantly, we confirmed that most of the cytoplasmic granules of Venus-FUS^wt^ and mutants are G3BP1-positive SGs under the cotreatment with CLM and NU7441 (Supplementary Figures 1A–C).

**Figure 2:**
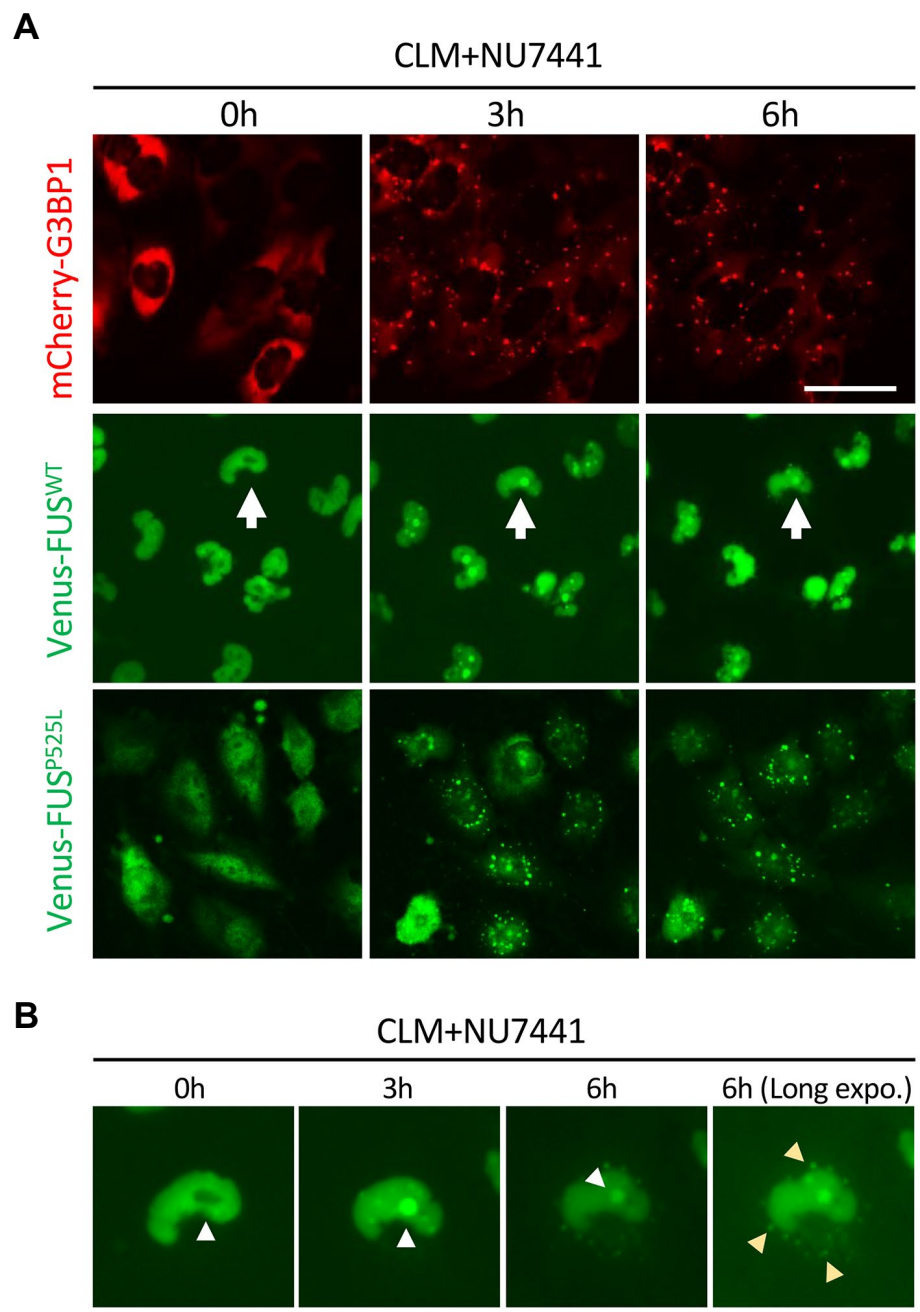
Live imaging of cells stably expressing mCherry-G3BP1, Venus-FUS^WT,^ and Venus-FUS^P525L^. **(A)** Cells were treated with 1 μM NU7441 for 3 h and were then treated with 100 nM CLM for 0, 3, and 6 h in the presence of 1 μM NU7441. To visualize cytosolic SGs, U251 MG (KO) cells overexpressing mCherry-G3BP1 were generated and treated with 100 nM CLM for 3 and 6 h. Scale bar: 50 μm. **(B)** The cells with white arrows in the images are shown at high magnification. The yellow arrowheads indicate SGs in the long-exposure image.

In a previous report, the topoisomerase I inhibitor camptothecin rapidly induced the translocation of FUS into the nucleolus (Martinez-Macias et al., 2019). We also observed the translocation of FUS into the nucleolus, which is a fibrillarin-positive nuclear structure, by a similar stimulus, i.e., the CLM-induced DNA damage response. Interestingly, this nucleolar localization was also observed in FUS^WT^, FUS^P525L^ and FUS^H517D^, suggesting the independence on FUS mutation (Figure 1D). To better understand the translocation mode of FUS proteins and SG formation, we conducted live cell imaging of Venus-FUS^WT^, Venus-FUS^P525L^, and mCherry-tagged G3BP1 (mCherry-G3BP1) proteins. SG formation was monitored *via* the subcellular localization of mCherry-G3BP1. Within 3 h after CLM treatment, formation of mCherry-G3BP1-positive cytosolic SGs was observed, but Venus-FUS^WT^ remained in the nucleus, although nucleolar accumulation of FUS was observed (Figure 2A). At 6 h after CLM treatment, Venus-FUS^WT^ was translocated into cytosolic SGs, while the nucleolar accumulation of FUS was relatively weakened (Figures 2A,B). These results mirrored the localization of endogenous FUS and another fusion tag protein FLAG-FUS^WT^, which was not translocated into cytosolic SGs within 3 h after treatment with CLM and NU7441 but was translocated at 6 h (Nogami et al., 2021a; Supplementary Figure 1B). On the other hand, Venus-FUS^P525L^ was partially translocated into cytosolic SGs at 3 h after cotreatment with CLM/NU7441 and fully translocated into cytosolic SGs at 6 h (Figure 2A). These observations suggest that G3BP1-positive SGs first form, and cytosolic nonphosphorylated FUS is then incorporated into G3BP1-positive cytosolic SGs during DNA damage stress under the impairment of DNA-PK activation by treatment with NU7441, indicating the unique feature of the FUS protein for FUS-SGs formation. In fact, we have obtained the consistent result that show the DNA-PK dependency by immunocytochemistry for endogenous FUS protein. On the other hands, another nuclear-enriched RBP, TDP-43 translocate to the cytoplasm and into SGs after stimulation with CLM alone, similar to G3BP1, while the localization of QKI5 does not change even under cotreatment with CLM/NU7441 for 6 h (Supplementary Figures 2A–C).

### A role of Ser/Thr residues in the N-terminal region of FUS in the protective effect against mislocalization to cytosolic SGs

**Figure 3:**
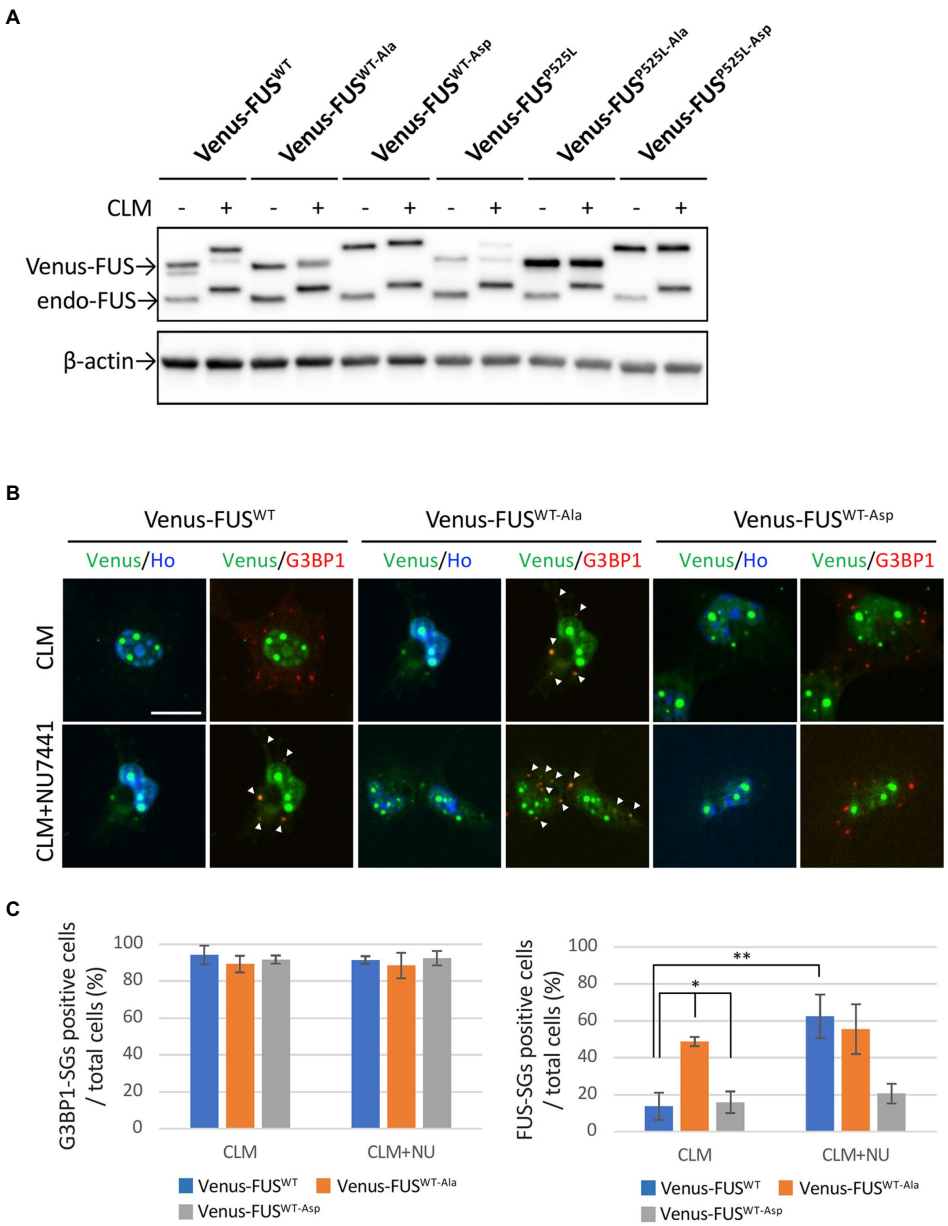
Effect of replacing 12 S/TQ residues with AQ or DQ residues in the N-terminal region of wild-type and mutant FUS proteins. **(A)** U251 MG (KO) cells stably overexpressing Venus-FUS^WT-Ala^, Venus-FUS^WT-Asp^, Venus-FUS^P525L-Ala,^ and Venus-FUS^P525L-Asp^ were cloned, and Western blotting with an anti-FUS antibody was performed to confirm the expression of Venus-tagged FUS and endogenous FUS proteins in each cell line from three biological replicates. Cells were treated with 100 nM CLM for 5 h. An anti-β-actin antibody was used as a control. **(B)** The subcellular distribution of Venus-FUS^WT^, Venus-FUS^WT-Ala^ and Venus-FUS^WT-Asp^ (Green) co-stained with G3BP1 (Red), a marker for SGs and Hoechst (Blue), a marker for nuclei was observed in cells by fluorescence microscopy. Cells were treated with 1 μM NU7441 for 1 h and were then treated with 100 nM CLM for 5 h in the presence of 1 μM NU7441 or DMSO (control). Arrowheads indicate G3BP1 positive cytosolic SGs with Venus-FUS^WT^ or Venus-FUS^WT-Ala^ proteins. Scale bar: 20 μm. **(C)** Quantitative data of the ratio of G3BP1-SGs-positive cells (Left) and FUS-positive SGs (Right) in total cells (at least 3 biological replicates; mean ± SD; *p < 0.001; Dunnett’s test and **p < 0.001 *t*-test).

We observed that Venus-FUS^P525L^ is more likely to mistranslocate into cytoplasmic SGs with CLM treatment alone and is less efficiently phosphorylated by DNA-PK than are Venus-FUS^WT^ and Venus-FUS^H517D^ (Figures 1A–C). Considering that 12 Ser/Thr residues in the N-terminal region of FUS are phosphorylated by DNA-PK (Deng et al., 2014; Monahan et al., 2017), we hypothesized that the impaired phosphorylation of FUS^P525L^ is the cause of FUS^P525L^ mislocalization into cytoplasmic SGs. To elucidate the roles of the 12 phosphorylation sites in FUS, we replaced these 12 Ser/Thr residues with Ala and Asp residues to generate DNA-PK–non-phosphorylatable forms and DNA-PK phospho-mimic forms, respectively, in Venus-FUS^WT^ and Venus-FUS^P525L^ (Supplementary Figure 3A). The Venus-FUS^WT-Ala^ and Venus-FUS^WT-Asp^ protein bands on the SDS–PAGE gels were consistent with the nonphosphorylated and phosphorylated forms of the Venus-FUS proteins, respectively (Figure 3A). Importantly, we confirmed that the band shift associated with CLM stimulated phosphorylation in both endogenous and exogenous FUS were cancelled by DNA-PK inhibitor NU7441, reflecting the previous reports from phos-tag gel shift assay to prove that the band shift of FUS is well correlated to phosphorylation levels (Rhoads et al., 2018; Nogami et al., 2021a; Supplementary Figure 3B). We found that Venus-FUS^WT-Asp^ was not localized into cytoplasmic SGs even after treatment with both CLM and NU7441. At the same time, Venus-FUS^WT-Ala^ was recruited into cytoplasmic SGs by treatment with CLM alone, indicating the unique feature of DNA-PK dependent FUS-SGs formation and suggesting the possibility that DNA-PK activity protects against recruitment into cytoplasmic SGs (Figures 3B,C). However, we did not observe differences in nucleolar accumulation in the FUS mutants with the replacement of the 12 Ser/Thr residues (Figure 3B).

**Figure 4:**
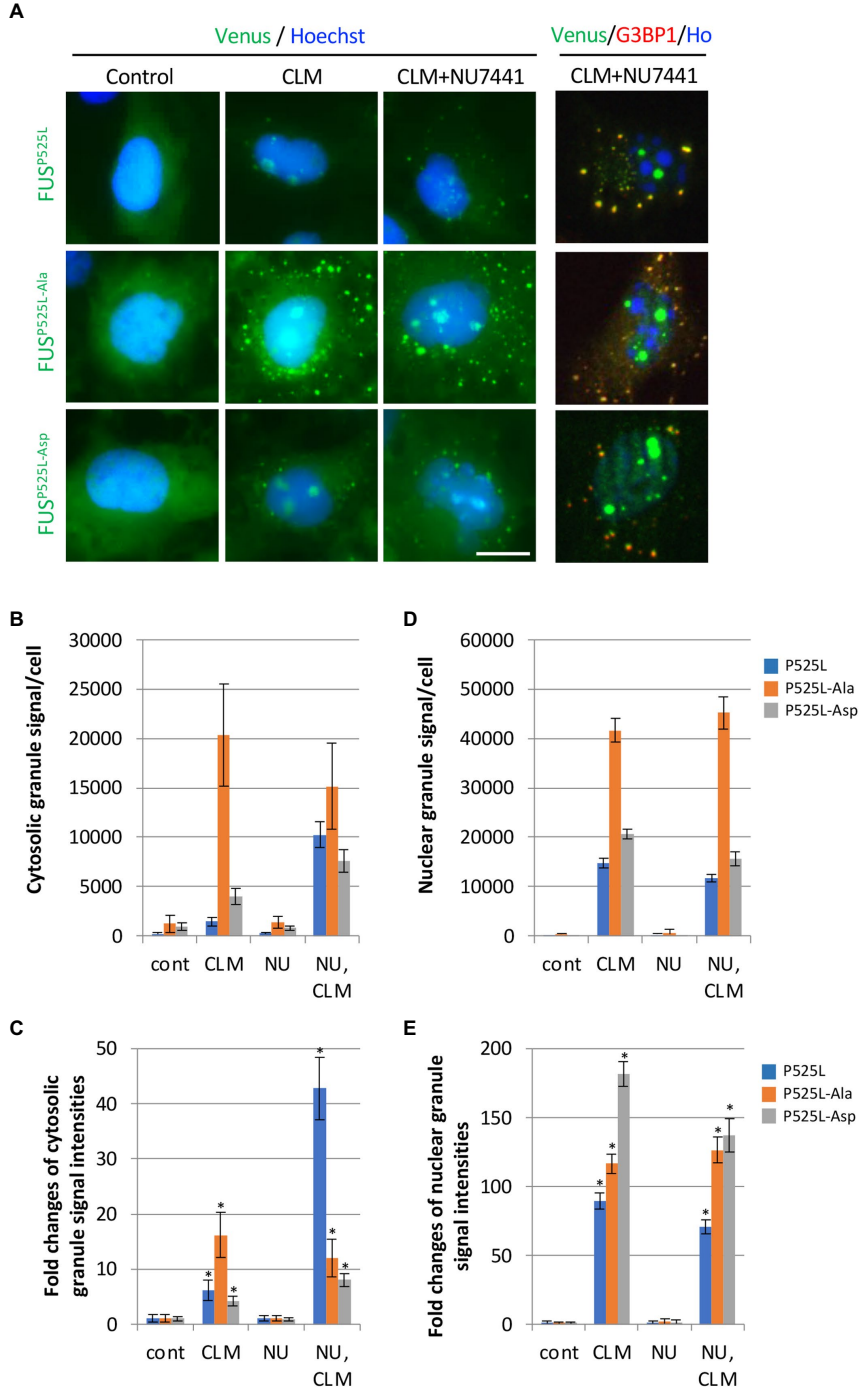
Synergistic effect of replacing 12 S/TQ residues with AQ or DQ residues in the N-terminal region of FUS^P525L^ on SG formation during CLM/NU7441 stimulation. **(A)** The subcellular distribution of Venus-FUS^P525L^, Venus-FUS^P525L-Ala,^ and Venus-FUS^P525L-Asp^ proteins co-stained with G3BP1 (Red), a marker for SGs and Hoechst (Blue), a marker for nuclei was observed by fluorescence microscopy. Cells were pretreated with 1 μM NU7441 and were then treated with 100 nM CLM in the presence of 1 μM NU7441 or DMSO (control) for 5 h. Scale bar: 20 μm. **(B,C)** Quantification of Venus fluorescence signals at cytosolic granules and fold changes of their signals to each control of Venus-FUS^P525L^, Venus-FUS^P525L-Ala^ and Venus-FUS^P525L-Asp^ with the IN Cell Analyzer imaging system. The result of statistical analysis is shown in Supplementary Table 1. **(D,E)** Quantification of Venus fluorescence signals at nuclear granules and fold changes of their signals to each control of Venus-FUS^P525L^, Venus-FUS^P525L-Ala^ and Venus-FUS^P525L-Asp^ with the IN Cell Analyzer imaging system. Asterisk indicates significant change relative to FUS derivates-SGs containing cells of DMSO control with CLM + NU7441 treatment (*t*-test *p* < 0.001). The result of statistical analysis is also shown in Supplementary Table 1.

We next investigated the effects of the 12 Ser/Thr phosphorylation sites in Venus-FUS^P525L^. Interestingly, FUS^P525L^ showed a synergistic increase in recruitment to cytoplasmic granules and the fluorescence intensity per cell from CLM stimulation alone to cotreatment with CLM and NU7441 (Figures 4A–C; Supplementary Table 1). We confirmed that FUS cytoplasmic granule co-stained with G3BP1 positive SGs. On the other hand, unlike the other mutants, FUS^P525L-Ala^ showed a marked increase in recruitment to cytoplasmic granules with CLM stimulation alone but did not show a synergistic increase in recruitment to SGs under cotreatment with CLM and NU7441. Although the Venus-FUS^P525L-Asp^ mutation failed to inhibit recruitment to cytoplasmic SGs, unlike in cells expressing Venus-FUS^WT-Asp^ (Figures 3B–C), the synergistic increase in recruitment to SGs by cotreatment with NU7441 was abolished (Figures 4A–C; Supplementary Table 1). Importantly, nucleolar accumulation of all FUS^P525L^ mutants was observed, but no synergistic increase by treatment with NU7441 was detected, indicating that nucleolar accumulation is not DNA-PK dependent (Figure 4A,D,E; Supplementary Table 1). These data suggest that DNA-PK plays a protective role against cytosolic mislocalization of FUS to SGs through phosphorylation of the FUS N-terminal domain during DNA damage stress.

### Compound screening

**Figure 5:**
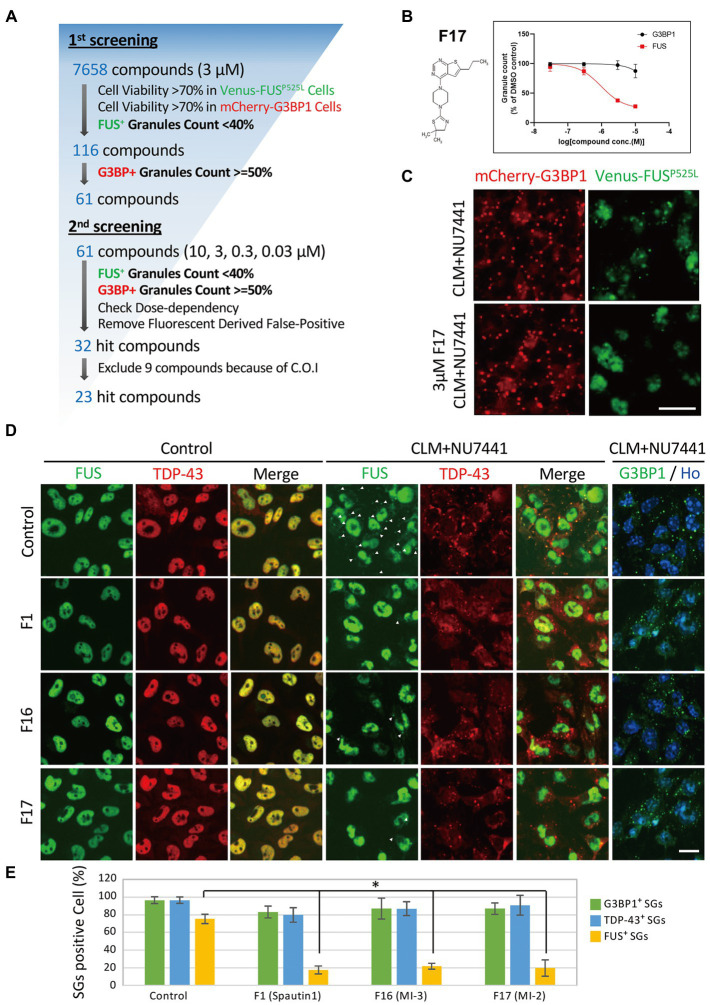
Compound screening for inhibition of FUS-containing SG formation. **(A)** Compound screening flowchart. See the methods section for details. **(B)** Dose-response curve of Compound F17 as one of the 23 hit compounds. T-3811487 (3 μM), a representative hit compound, did not inhibit the formation of G3BP1-positive SGs (black line) but inhibited the localization of FUS^P525L^ (red line) to SGs. The vertical axis shows the inhibition rate (%) of granule formation. **(C)** Fluorescence image of cells treated with 3 μM F17. The scale bars represent 20 μm. **(D)** Validation of the effects of the hit compounds on endogenous FUS protein localization. U251MG (KO) cells were pretreated with 3 μM F1, F16, and F17 and were then treated with 100 nM CLM and 10 μM NU7441 or with DMSO (control) for 6 h. The images show immunocytochemistry for G3BP1-positive puncta G3BP1 (green) and Hoechst (blue), control for SGs, TDP-43 (red), and FUS (green). The arrowheads indicate FUS-positive SGs. The scale bars represent 20 μm. **(E)** Relative population of the cells containing cytosolic stress granules were calculated to total cells. Values represent mean ± SD. Asterisk indicates significant change relative to FUS-SGs containing cells of DMSO control with CLM + NU7441 treatment (*t*-test *p* < 0.001).

**Figure 6:**
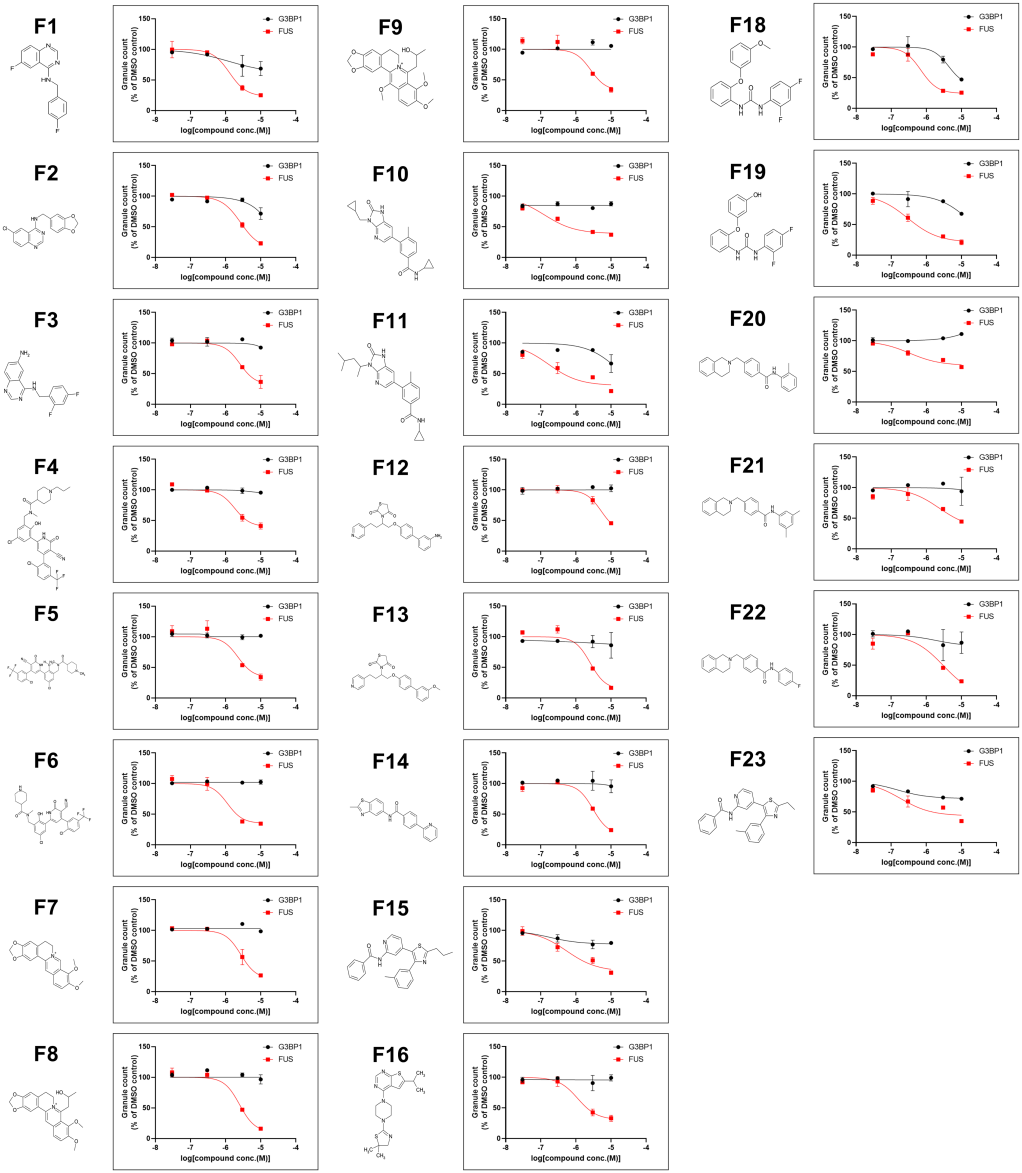
A list of 23 hit compounds that inhibit FUS-positive SG formation. Structures of the 23 hit compounds except for F17 are shown in Figure 5B and dose-response curves for reduced mislocalization of FUS^P525L^ at cytosolic SGs.

We next performed a compound screen to identify compounds that can inhibit CLM-and NU7441-induced formation of FUS^P525L^-positive SGs without changing the formation of G3BP1-positive SGs as the 1st screen using mCherry-G3BP1-and Venus-FUS^P525L^-expressing U251 MG (KO) astrocytoma cell lines. In the 1st screening, the 7,658 compounds were evaluated at a single concentration (3 μM). To eliminate compounds that were toxic to cells, compounds leaving at least 70% more viable cells than observed in the control cell group were selected based on cell counts from images of nuclear Hoechst staining in both Venus-FUS^P525L^-expressing and mCherry-G3BP1-expressing cells (Figure 5A). Next, cytoplasmic Venus-FUS puncta that appeared under CLM and NU7441 stimulation were quantified, and compounds for which the number of puncta was reduced by 40% or less compared to that in control cells were selected (116 compounds; Figure 5A). To identify compounds that reduced the number of FUS puncta without changing the number of SGs, we quantified cytoplasmic mCherry-G3BP1 puncta that appeared under CLM and NU7441 stimulation and selected compounds that reduced the number of puncta to 50% or more compared to that in control cells (61 compounds; Figure 5A). In the 2nd screen, to check the reproducibility of the 61 hit compounds from the 1st screening, 303 compounds, including the compounds adjacent to the 61 hits, were evaluated at 4 concentrations (10, 3, 0.3, and 0.03 μM). To exclude false-positives, the compounds showing concentration dependency with a low signal-to-noise ratio were selected from the compounds that reduced the FUS-positive SG count without reducing the G3BP1 SG count, as in the 1st screen. Finally, we obtained 23 compounds as hit compounds (Figure 5A). The representative compound F-17, showing a typical trend, was a commercially available compound—MI-2, also known to be a Menin-MLL interaction inhibitor (Figures 5B,C). Importantly, we confirmed that compound F17 did not affect cell viability at any concentration under the CLM and NU7441 stimulation (Supplementary Figures 4A,B). We next tested whether these compounds truly inhibit the localization of not only exogenous FUS^P525L^-SGs but also endogenous FUS protein to SGs. First, we confirmed that endogenous FUS also localized in mCherry-G3BP1-positive stress granules upon cotreatment with CLM and NU7441 (Supplementary Figure 1C). We used three commercially available compounds—spautin-1 (F1), known to be an autophagy inhibitor *via* targeting two ubiquitin-specific peptidases (USP-10 and USP-13) (Liu et al., 2011), the Menin-MLL interaction inhibitor (Grembecka et al., 2012) MI-2 (F17), and MI-3 (F16)—and observed their effects on endogenous FUS localization into SGs. Pretreatment with any of the three compounds (3 μM) obviously inhibited the localization of endogenous FUS to SGs without altering the localization of G3BP1 or TDP-43 proteins to SGs, indicating the validity of our compound screen (Figures 5D,E). In addition, treatment with these three compounds did not alter the protein level of endogenous FUS itself (Supplementary Figure 5). Furthermore, we examined FUS into SGs inhibitory activity in the ALS-linked mutations of FUS using two compounds, F1 and F17, which is the same molecular target with F16 since mutant FUS proteins have been shown to localize SGs due to oxidative stress, such as arsenite unlike wild-type FUS proteins (Supplementary Figure 6A). However, no inhibitory activity of mutant FUS into SGs was observed, possibly due to the different molecular pathways do not DNA damage response (Supplementary Figure 6B,C). Finally, we added the list of hit compounds, including graphs showing the concentration dependence of the counts of FUS-and G3BP1-positive SGs and their IC50 (μM) of the number of FUS-positive SGs with compounds structure (Figure 6; Supplementary Table 2).

## Discussion

We previously reported the localization of the FUS protein to cytoplasmic SGs by a combination of DNA damage induction and treatment with an inhibitor of DNA-PK (Nogami et al., 2021a). In the present study, we clarified the requirement for DNA-PK activity in the localization of FUS to cytoplasmic SGs in detail by using Venus-tagged FUS proteins carrying ALS-linked mutations and mutations in the sites of DNA-PK–regulated phosphorylation. Using this system, we also screened small molecule compounds that inhibited FUS localization but not G3BP1 to cytoplasmic SGs. As a result, we identified small molecule compounds with activities such as protein kinase inhibition and chromatin regulation. The effects of these compounds on the translocation of endogenous FUS protein were also validated (Figures 5, 6).

SGs are structural membraneless organelles that are thought to be one of the cellular defense components essential for maintaining cellular function. SGs contain mainly mRNAs and cytoplasmic RNA-binding proteins; for example, G3BP1 is a major marker protein (Gomes and Shorter, 2019). SGs can divide specific sub compartments, cores versus shells. G3BP1 exists in SGs cores and is a key protein of SGs formation. Upon stress, ALS-associated RBPs, such as FUS and TDP-43, may contribute towards formation of SGs shell component, which is sensitive to RNase treatment (Fang et al., 2019). Previous report have indicated that siFUS KD, but not siTDP-43, did not affect the SGs assembly (Aulas et al., 2012). In our cell system, the nuclear-enriched RBP FUS does not localize to SGs and affect SGs formation in response to arsenite stimulation, suggesting FUS does not play a role of SGs formation. On the other hand, the C-terminal mutants of FUS, such as FUS^H517D^, FUS^R521C^, and FUS^P525L^, which harbor various mutations in FUS identified as ALS-linked mutations, show evident recruitment to SGs under stress conditions (Higelin et al., 2016; Ichiyanagi et al., 2016; Supplementary Figure 6A). This could be due to cytoplasmic leakage of the mutated FUS protein itself, caused by its weakened nuclear translocation signal. It has also been reported that the cytoplasmic localization of these three mutants correlates with the severity of neurodegeneration in ALS, suggesting a risk of cytoplasmic leakage (Dormann et al., 2010). Therefore, the localization of insoluble and aggregating molecules such as FUS in SGs is considered a risk factor for neurodegeneration, as opposed to SG formation as an intrinsic stress defense function. Modifying the FUS translocation to SG without inhibiting SGs assembly by small molecules might be useful like a previous study for TDP-43 protein (Fang et al., 2019). However, we could not deny the possibility of secondary effects by the compounds, including inhibition of unknown function of wild type FUS protein and nuclear-retained mutated FUS proteins in SGs. Other groups and we have reported that the endogenous FUS migrates to SGs in response to DNA damage stress mediated by topoisomerase I induction or CLM stimulation (Rhoads et al., 2018; Martinez-Macias et al., 2019; Nogami et al., 2021a). In particular, we argue that the translocation of endogenous FUS to SGs is dependent on DNA-PK activity (Figure 3; Nogami et al., 2021a). Although ATM pathway, which is another down stream of DNA damage response, have not been considered in this study, we believe that DNA-PK activity plays important roles in FUS cellular dynamics as evidenced by the solubility of phospho-mimic mutant FUS protein (Supplementary Figure 7A). In contrast, the other nuclear-enriched RBPs, TDP-43 and QKI5 behaved differently under DNA damage stress conditions, showing no DNA-PK dependence (Supplementary Figure 2). While these three proteins show broadly similar nuclear subcellular localization under unstressed conditions, TDP-43 is concentrated in the nucleus at Gem, and nuclear speckles (Tsuiji et al., 2013), and QKI5 is localized at nuclear speckles and regulates pre-mRNA splicing in neural stem cells (Hayakawa-Yano et al., 2017). The difference in the mechanism of subcellular translocation of these three nuclear RBP families might be due to their distinct NLS types and protein modifications (Wu et al., 1999; Lee et al., 2006; Hofweber et al., 2018; François-Moutal et al., 2019). Given the above observations, the activation of DNA-PK and the control of the corresponding signaling pathway by the compounds found in this study might provide critical tools to mitigate FUS-specific condensation and aggregation, thereby ameliorating FUS-ALS pathology.

In this study, we found that the FUS protein rapidly localizes to the nucleolar cap before the formation of FUS-positive cytoplasmic granules. The localization of endogenous and Venus-tagged FUS to the nucleolus, specifically in response to topoisomerase I-induced DNA cleavage, has recently been reported (Martinez-Macias et al., 2019). Alternatively, it has been reported that transcription-arresting agents also induce FUS accumulation in pol-II DNA damage foci, and this accumulation could be involved in the prevention or repair of R-loop–associated DNA damage (Hill et al., 2016). Interestingly, our observations indicate that FUS does not localize to the nucleolus under steady-state conditions but rapidly changes its localization to the nucleolus upon induction of DNA damage by CLM (Figure 1D) and that when the localization of FUS to cytoplasmic SGs occurs, the localization of FUS to the nucleolus is decreased (Figure 2B). Our analysis did not clarify whether the FUS localized to cytoplasmic SGs originates from the nucleolar cap during CLM/NU7441 stimulation. On the other hand, we found that this localization of FUS to SGs was synergistically increased by cotreatment with CLM and NU7441; in contrast, the translocation of FUS to the nucleolus did not show a synergistic response, suggesting that the mechanism of nucleolar localization is independent of DNA-PK activity (Figures 4D,E). In the future, further analysis, including the various compounds that we identified in this study, will clarify the mechanisms involved in the translocation of FUS from the nucleolar cap and SG formation.

Among the 23 compounds, we found several compounds that act on p38 MAP kinase pathways in this study. Significantly, the general p38 MAP kinase inhibitor SB203580 also inhibited the localization of endogenous FUS to SGs (Supplementary Figure 7B). In addition, F17, listed as an example of a representative compound in Figure 5, was a Menin-MLL interaction inhibitor; this compound is also called MI-2 and is a commercial compound. Importantly, MI-3, which also showed a similar action, was also identified as a hit compound; therefore, this chromatin-regulating pathway is thought to be involved in the inhibition of FUS translocation. Both of these compounds, as expected, inhibited the formation of FUS-positive SGs under CLM/NU7441 cotreatment (Figures 5D,E). Although the present U251 cellular model is actually a limitation in this study, we would like to extend such effects to the 23 other compounds and validate them by using previous our iPS-derived motor neuron models in the future. Another significant compound is F7, berberine chloride, which is an alkaloid also known to be a ligand of DNA/RNA G-quadruplexes (Che et al., 2018; Dumas et al., 2021). Recently, the interaction between FUS/FUS-ALS mutations and G4-DNA/RNA was well characterized based on G4-RNA–dependent LLPS and liquid-to-solid transition *in vitro* (Ishiguro et al., 2021a,b). In both *in vitro* and *in vivo* systems, the roles of FUS, G4-RNA interactions, and G4-RNA ligands in SG and aggregate formation will be further studied in the future. These results suggest that it must be feasible to enrich target candidates in screens using our biologically annotated library and conventional chemical screens. However, we did not find a common biological pathway to which all hit compounds directly correlate, suggesting that each compound acts to mitigate the formation of FUS-SGs through different molecular pathways. Therefore, these hit compounds can be used as tools to elucidate the mechanisms of FUS localization to several intracellular membraneless organelles and FUS protein aggregation. Finally, we hope that these compounds will help to elucidate the FUS aggregation mechanism and the etiological mechanisms of ALS and neurodegeneration.

## Data availability statement

The raw data supporting the conclusions of this article will be made available by the authors, without undue reservation.

## Author contributions

MN, HO, and MY: conceptualization. MN, OS, KA-T, YH-Y, and TF: data curation. MN, OS, and MY: formal analysis. MY and HO: funding acquisition. KO, HI, MY, and HO: project administration. MN, OS, HI, and MY: resources. MY and HO: supervision. MN, KO, HO, and MY: validation. MN, OS, and MY: visualization. MY and MN: writing – original draft. MY, MN, YH-Y, and HO: writing – review and editing. All authors contributed to the article and approved the submitted version.

## Funding

This work was supported by grants from the SIL Research Fund from Takeda Pharmaceutical Company, Ltd., Japan and a Grant-in-Aid for Scientific Research from the JSPS (grant number JP20H00485a) to MY and HO. This work is also supported by a Grant-in-Aid for Transformative Research Areas (A) from The MEXT (grant number JP22H05589) and a Grant-in-Aid for Scientific Research from the JSPS (grant number P19H03543), the Takeda Science Foundation, Japan, Takeda-COCKPI-T and the SERIKA Fund to MY the Takeda Science Foundation and the ALS Foundation (Japan ALS Association) to YH-Y.

## Conflict of interest

HO is a paid member of the Scientific Advisory Board of San Bio Co., Ltd., and K Pharma, Inc. MY is a scientific advisor of K Pharma, Inc.

MN, OS, KA-T, KO, and HI were employed by the company Takeda Pharmaceutical Company Limited.

The remaining authors declare that the research was conducted in the absence of any commercial or financial relationships that could be construed as a potential conflict of interest.

## Publisher’s note

All claims expressed in this article are solely those of the authors and do not necessarily represent those of their affiliated organizations, or those of the publisher, the editors and the reviewers. Any product that may be evaluated in this article, or claim that may be made by its manufacturer, is not guaranteed or endorsed by the publisher.

## Abbreviations

FUS: Fused in sarcoma/translated in liposarcoma
SGs: Stress granules
ALS: Amyotrophic lateral sclerosis
RBP: RNA binding protein
LLPS: Liquid-liquid phase separation
CLM: Calicheamicin
