# Supplementary material for: DNA damage stress-induced translocation of mutant FUS proteins into cytosolic granules and screening for translocation inhibitors
Source: Front Mol Neurosci. 2022 Dec 20;15:953365. doi: 10.3389/fnmol.2022.953365 (PMC9808394; doi:10.3389/fnmol.2022.953365)
Supplement: Supplementary file 2 [file Data_Sheet_1.pdf]

TITLE

DNA damage stress-induced translocation of mutant FUS proteins into cytosolic granules and screening for translocation inhibitors

Masahiro Nogami[1,2*#], Osamu Sano[1#], Keiko Adachi-Tominari[1], Yoshika Hayakawa-Yano[3,4], Takako Furukawa[4], Hidehisa Iwata[1], Kazuhiro Ogi[1,2], Hideyuki Okano[3] and Masato Yano[3,4,5*#]

[1]Innovative Biology Laboratories, Neuroscience Drug Discovery Unit, Research, Takeda Pharmaceutical Company Limited, Fujisawa 251-8555, Japan

[2]Shonan Incubation Laboratories, Research, Takeda Pharmaceutical Company Limited, Fujisawa 251-8555, Japan

[3]Department of Physiology, School of Medicine, Keio University, Tokyo 160-8582, Japan

[4]Division of Neurobiology and Anatomy, Graduate School of Medical and Dental Sciences, Niigata University, Niigata 951-8510, Japan

[5]Lead Contact

#Equally contributed to this work

*Correspondence: masahiro.nogami@takeda.com, myano@med.niigata-u.ac.jp

CONTENTS

・Supplementary Figure 1-7

・Supplementary Table 1-2 and Figure 1-7 Legends

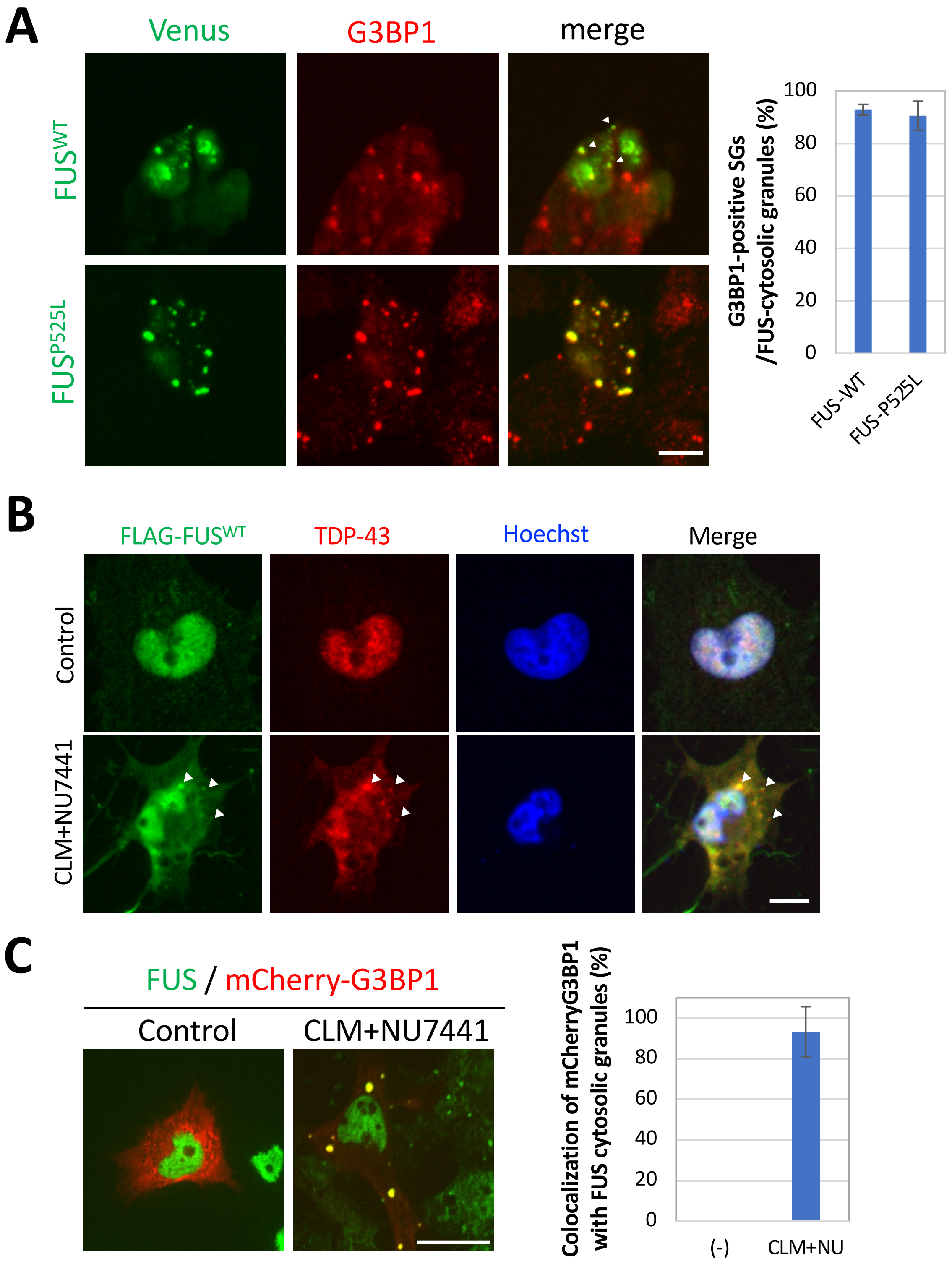

Supplemental Figure 1.

Nogami et al.

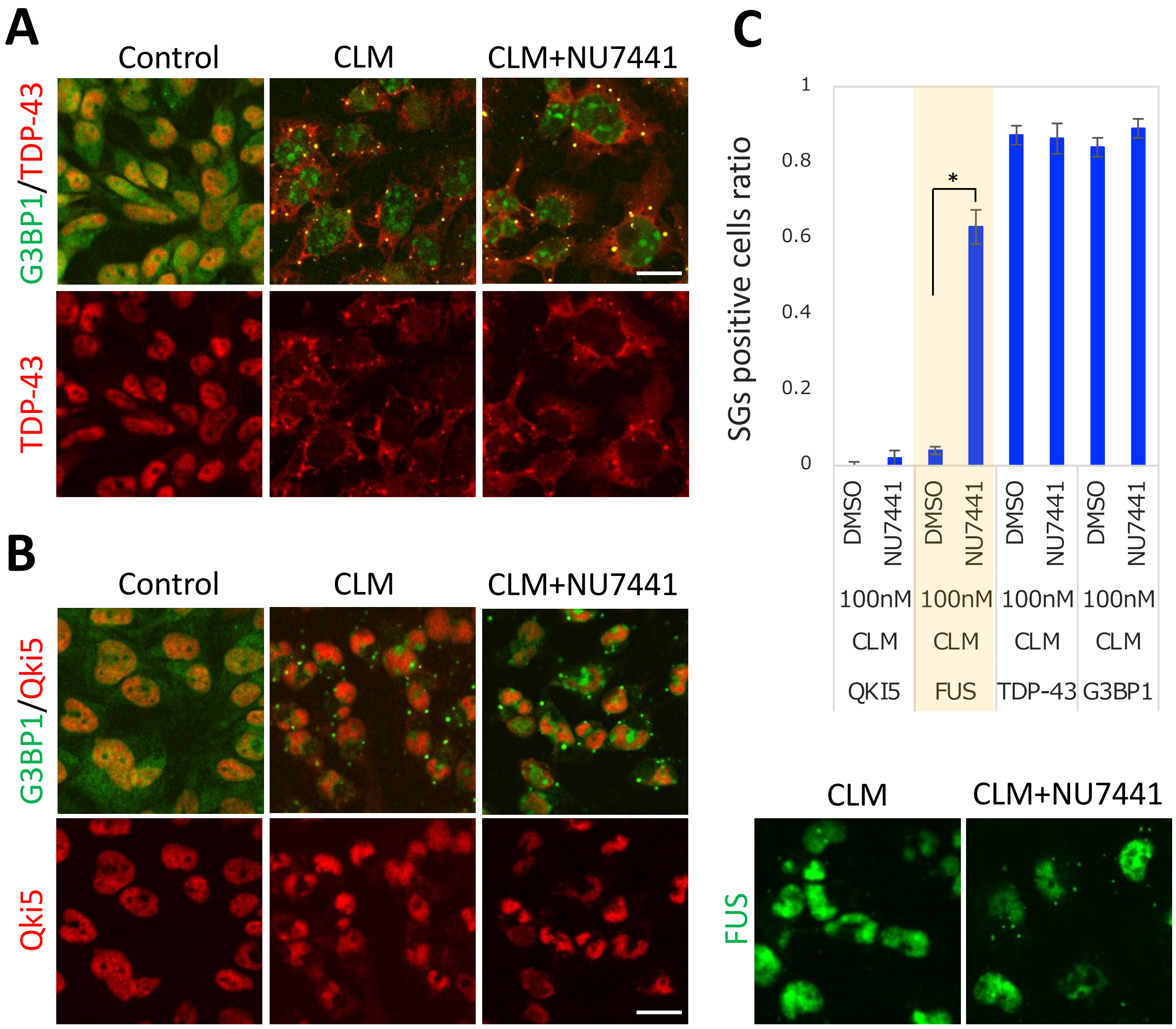

Supplemental Figure 2.

Nogami et al.

A

FUS^{WT}

ATGGCCTCAAACGATTATACCCAACAAGCAACCCAAAGCTATGGGGCCTACCCCACCCAGCCCGGGCAGGGCTATTCCCA
GCAGAGCAGTCAGCCCTACGGACAGCAGAGTTACAGTGGTTATAGCCAGTCCACGGACACTTCAGGCTATGGCCAGAGCA
GCTATTCTTCTTATGGCCAGAGCCAGAACACAGGCTATGGAACTCAGTCAACTCCCCAGGGATATGGCTCGACTGGCGGC
TATGGCAGTAGCCAGAGCTCCCAATCGTCTTACGGGCAGCAGTCCTCCTACCCTGGCTATGGCCAGCAGCCAGCTCCCAG
CAGCACCTCGGGAAGTTACGGTAGCAGTTCTCAGAGCAGCAGCTATGGGCAGCCCCAGAGTGGGAGCTACAGCCAGCAGC
CTAGCTATGGTGGACAGCAG

FUS^{Ala}

ATGGCCTCAAACGATTATGCCCAACAAGCAGCCCAAAGCTATGGGGCCTACCCCGCCCAGCCCGGGCAGGGCTATGCCCA
GCAGAGCGCTCAGCCCTACGGACAGCAGAGTTACAGTGGTTATGCCCAGTCCACGGACACTTCAGGCTATGGCCAGAGCA
GCTATTCTTCTTATGGCCAGGCCCAGAACACAGGCTATGGAGCTCAGTCAACTCCCCAGGGATATGGCTCGACTGGCGGC
TATGGCAGTGCCCAGAGCGCCCAATCGTCTTACGGGCAGCAGTCCTCCTACCCTGGCTATGGCCAGCAGCCAGCTCCCAG
CAGCACCTCGGGAAGTTACGGTAGCAGTGCTCAGAGCAGCAGCTATGGGCAGCCCCAGAGTGGGAGCTACGCCCAGCAGC
CTAGCTATGGTGGACAGCAG

FUS^{Asp}

ATGGCCTCAAACGATTATGACCAACAAGCAGACCAAAGCTATGGGGCCTACCCCGACCAGCCCGGGCAGGGCTATGACCA
GCAGAGCGATCAGCCCTACGGACAGCAGAGTTACAGTGGTTATGACCAGTCCACGGACACTTCAGGCTATGGCCAGAGCA
GCTATTCTTCTTATGGCCAGGACCAGAACACAGGCTATGGAGATCAGTCAACTCCCCAGGGATATGGCTCGACTGGCGGC
TATGGCAGTGACCAGAGCGACCAATCGTCTTACGGGCAGCAGTCCTCCTACCCTGGCTATGGCCAGCAGCCAGCTCCCAG
CAGCACCTCGGGAAGTTACGGTAGCAGTGATCAGAGCAGCAGCTATGGGCAGCCCCAGAGTGGGAGCTACGACCAGCAGC
CTAGCTATGGTGGACAGCAG

B

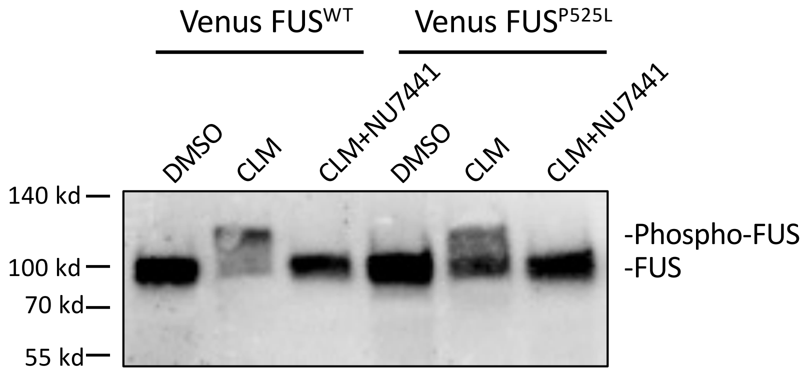

Supplemental Figure 3.

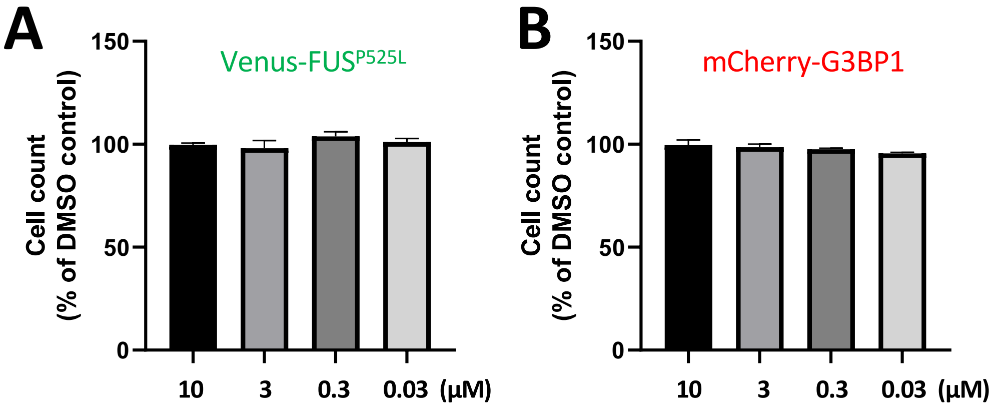

Supplemental Figure 4.

Nogami et al.

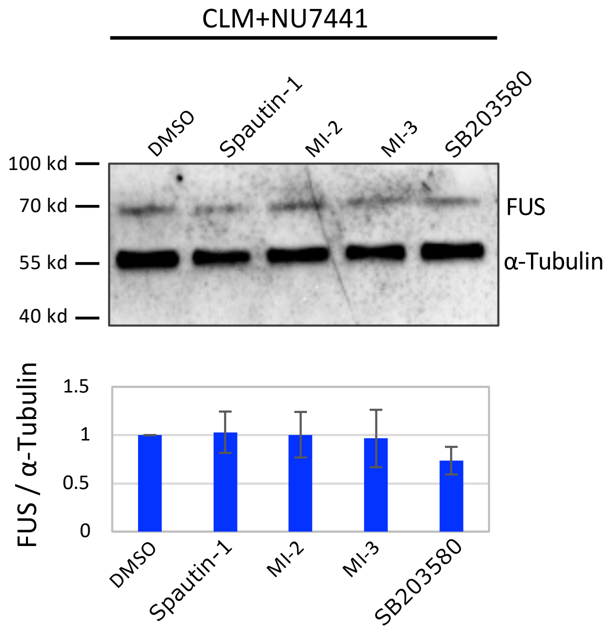

Supplemental Figure 5.

Nogami et al.

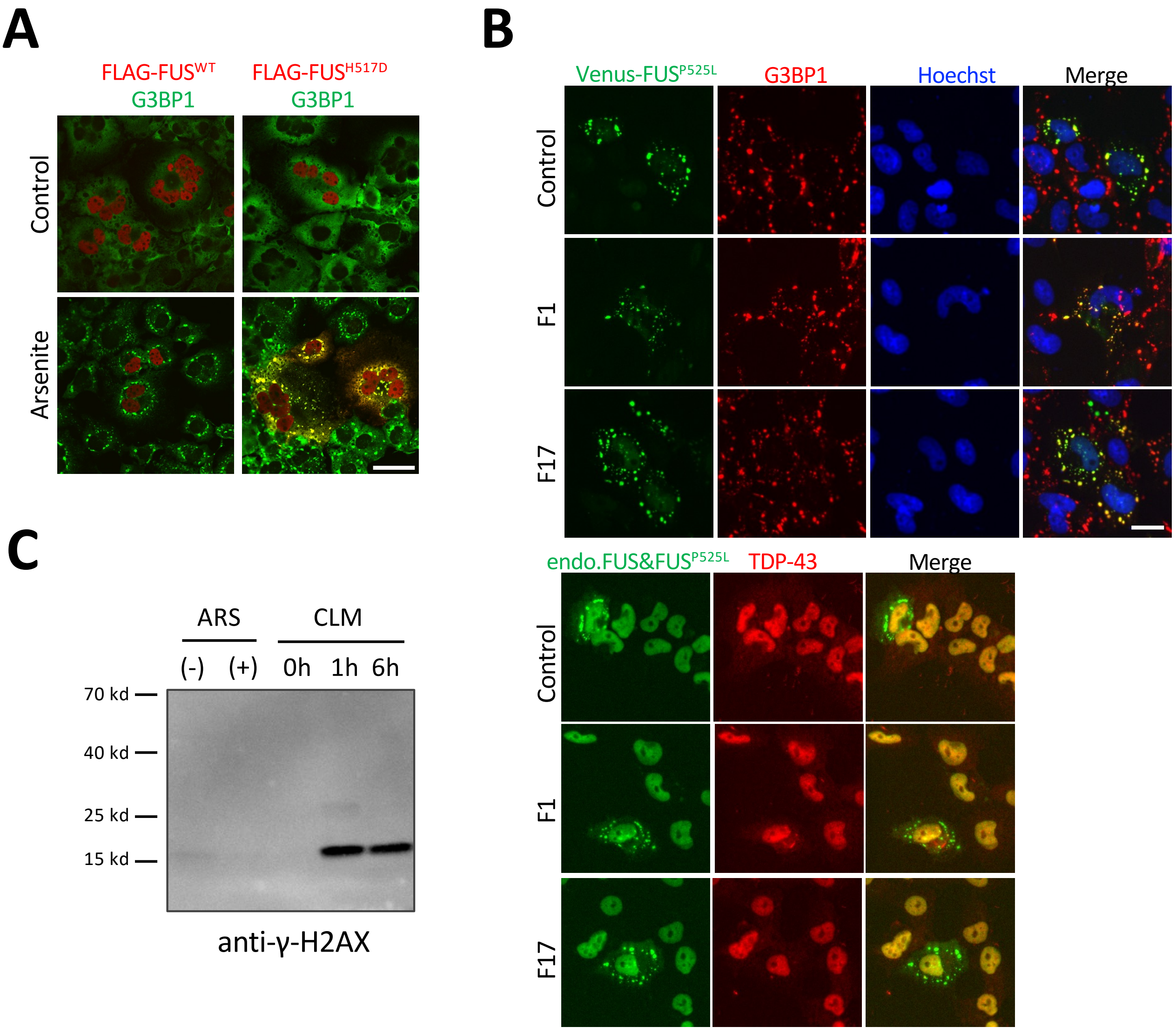

Supplemental Figure 6.

Nogami et al.

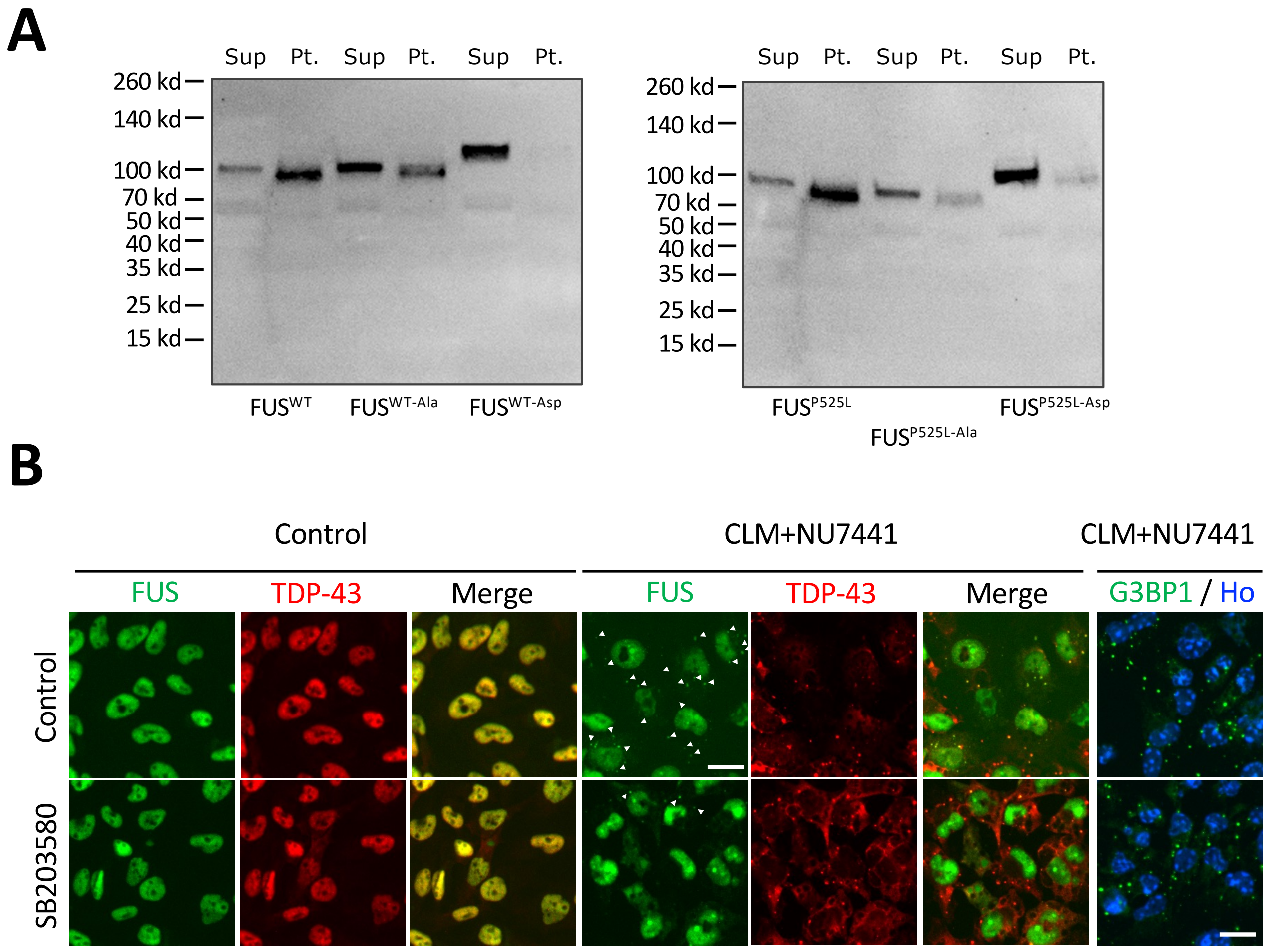

Supplemental Figure 7.

Nogami et al.

Supplementary Table 1. Signal intensities and statistical analysis for DNA-PK dependent SGs formation of FUS

Average and Standard deviations of total vesicle signal per cell in each U251MG(KO) cell lines are shown as cytoplasmic and nuclear, respectively. And the corresponding p-value are shown for each combination, calculated by two-tailed Student's t-test.

Supplementary Table 2. Hit compounds list

The 23 hit compounds are listed in the table with their IC_{50} values for reduced mislocalization of FUS^{P525L} into cytosolic SGs.

Supplementary Figure 1. Co-localization of cytoplasmic FUS granule with Stress granule proteins after CLM/NU7441 treatment

(A) Subcellular localization of Venus-FUS^{WT} and Venus-FUS^{P525L} and endogenous G3BP1 proteins in U251MG (KO) cells during CLM/NU7441 treatment. The scale bar indicates 10 μm. At least four fields of cells for each condition were imaged and the number of G3BP1 positive SGs among FUS-positive cytosolic SGs were counted. The ratio of co-localization with G3BP and FUS-SGs were calculated. Quantitative result indicates that almost FUS-cytosolic granules were co-stained with G3BP1 positive cells.

(B) Subcellular localization of N-terminal FLAG tagged FUS^{WT} and endogenous TDP-43 proteins in U251MG (KO) cells after CLM/NU7441 treatment and normal condition. The scale bar indicates 10 μm.

(C) Subcellular localization of endogenous FUS and mCherry-G3BP1 proteins in U251MG (KO) cells during CLM/NU7441 treatment. The scale bar indicates 20 μm. At least four fields of cells for each condition were imaged and the number of mCherry-G3BP1 positive SGs among FUS-positive cytosolic SGs were counted. The ratio of co-localization with mCherry-G3BP and FUS-SGs were calculated. Quantitative result indicates that almost FUS-cytosolic granules were co-stained with G3BP1 positive cells.

Supplementary Figure 2. Nuclear-enriched RBPs, TDP-43, and Qki5 after CLM and NU7441 treatment.

(A-B) The subcellular distribution of TDP-43 (A, red), Qki5 (B, red), and G3BP1 (green) proteins was observed by fluorescence microscopy. U251MG (KO) cells were treated with 100 nM CLM alone, 100 nM CLM/10 μM NU7441 or DMSO (control) for 6 hours. Scale bar: 20 μm

(C) Image for endogenous FUS proteins with CLM and CLM/NU7441 treatment. The graph indicates the quantitative data of the ratio of each RBPs positive cytosolic granules in total cells (at least 3 biological replicates; mean ± SD; *$p < 0.001$ t-test).

Supplementary Figure 3. Sequences of the FUS N-terminal region and DNA-PK dependent band shift of FUS protein.

(A) cDNA sequences of the FUS N-terminal region (140 amino acids/420 nucleotides downstream of the human FUS start codon). The 12 codons encoding S or T are shown in red. The codons encoding Q residues are shown in purple. The substituted nucleotides are shown in green. The non-phosphorylatable FUS mutant (FUS^{Ala}) with the replacement of all 12 S/T residues with A residues is shown in the middle. The phosphomimetic FUS mutant (FUS^{Asp}) with replacement of all 12 S/T residues with D residues is shown at the bottom.

(B) U251 MG (KO) cells, transiently expressing Venus FUS^{WT} and Venus FUS^{P525L} were pre-treated with control DMSO or 10 μM NU7441 for 3 h and then treated with control DMSO or 100 nM CLM for 6 h. Western blotting analysis was performed with anti-GFP antibody.

Supplementary Figure 4. Cell viability assay for compound screening

(A-B) Validation of the viability of Venus-FUS^{P525L}- and mCherry-G3BP1-expressing U251MG (KO) cells under the CLM and NU7441 co-treatment. A representative graph of the effects of Compound F17 at several concentrations is shown.

Supplementary Figure 5. Endogenous FUS protein level by compounds treatment

U251MG (KO) cells were pretreated with 3 μM Spautin1, MI-2, and MI-3 and were then treated with 100 nM CLM and 10 μM NU7441 or with DMSO (control) for 6 hours. Western blotting analysis was performed with anti-FUS antibody.

Supplementary Figure 6. Arsenite induced SGs formation of ALS-linked mutation of FUS

(A) Cos-7 cells, transiently expressing FLAG-FUS^{WT} and FUS^{H517D} were treated with 0.5 mM Arsenite (ARS) for 1 hour. Immunocytochemistyr was performed with anti-FLAG and anti-G3BP1 antibodies.

(B) U251MG (KO) cells, transiently expressing Venus-FUS^{P525L} were pretreated with 3 μM F1(Spautin1), F17(MI-2) and were then treated with 0.5 mM Arsenite (ARS) for 1 hour. Immunocytochemisty was performed with anti-G3BP1 and anti-TDP-43 antibodies.

(C) U251MG (KO) cells, transiently expressing Venus-FUS^{P525L} were pretreated with 3 μM F1(Spautin1), F17(MI-2) and were then treated with 0.5 mM Arsenite (ARS) for 1 hour or CLM for 0, 1 6 hours. Western blotting analysis was performed with anti-γH2AX antibody.

Supplementary Figure 7. Phospho-mimic mutation of FUS shows increased solubility to 1% triton / SB203580 and a p38 MAP kinase inhibitor, reduced FUS-positive SG formation.

(A) 293T cells were transiently expressed for Venus-FUS non-phosphorylatable and mimic mutants and cell extracts were fractionated into two fractions, 1% triton soluble or insoluble. then Western blotting analysis was performed with anti-GFP antibody.
(B) U251MG (KO) cells were pretreated with 3 μM SB204580, a p38 MAP kinase inhibitor, and were then treated with 100 nM CLM and 10 μM NU7441 or with DMSO (control) for 6 hours. The images show immunocytochemistry for G3BP1-positive puncta (green) as a positive control for SGs, TDP-43 (red), and FUS (green). The arrows indicate FUS-positive SGs. The scale bars represent 20 μm.
